# Rotary mechanism of V/A-ATPases—how is ATP hydrolysis converted into a mechanical step rotation in rotary ATPases?

**DOI:** 10.3389/fmolb.2023.1176114

**Published:** 2023-04-24

**Authors:** Ken Yokoyama

**Affiliations:** Department of Molecular Biosciences, Kyoto Sangyo University, Kyoto, Japan

**Keywords:** ATP synthase, V-ATPase, rotary motor protein, molecular motor, FOF1 ATP synthase

## Abstract

V/A-ATPase is a rotary molecular motor protein that produces ATP through the rotation of its central rotor. The soluble part of this protein, the V_1_ domain, rotates upon ATP hydrolysis. However, the mechanism by which ATP hydrolysis in the V_1_ domain couples with the mechanical rotation of the rotor is still unclear. Cryo-EM snapshot analysis of V/A-ATPase indicated that three independent and simultaneous catalytic events occurred at the three catalytic dimers (AB_open_, AB_semi_, and AB_closed_), leading to a 120° rotation of the central rotor. Besides the closing motion caused by ATP bound to AB_open_, the hydrolysis of ATP bound to AB_semi_ drives the 120° step. Our recent time-resolved cryo-EM snapshot analysis provides further evidence for this model. This review aimed to provide a comprehensive overview of the structure and function of V/A-ATPase from a thermophilic bacterium, one of the most well-studied rotary ATPases to date.

## Introduction

**FIGURE 1:**
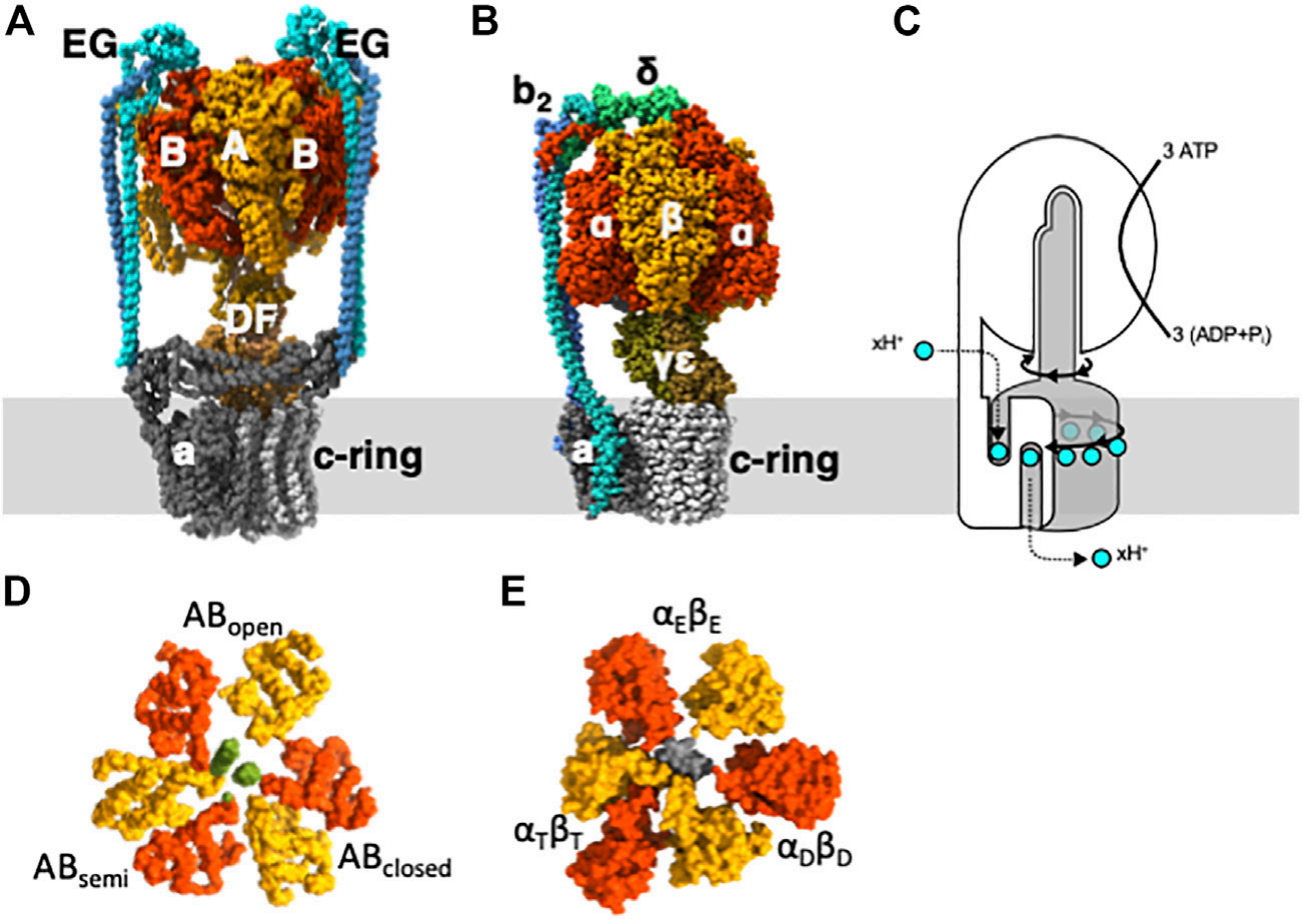
Subunit structure and function for V-type and F-type ATPases. **(A)** V/A-ATPase from *Thermus. thermophilus*. **(B)** bacterial F_o_F_1_ ATPase from *Geobacillus stearothermophilus*. **(C)** The schematic representation illustrates the rotary catalytic mechanism of rotary ATPases, where the rotation of the central rotor complex (grey) driven by ATP in the surrounding stator apparatus (white) rotates the membrane-embedded c-ring, leading to the translocation of protons across the membrane. Slice view of the C-terminal domain of V_1_ **(D)** and F_1_ **(E)**.

ATP synthases are enzymes that play crucial roles in energy metabolism (Boyer, 1997; Yoshida et al., 2001; Kuhlbrandt, 2019; Frasch et al., 2022). They convert an electrochemical proton motive force across the membrane generated by respiration into chemical energy that is stored in the form of ATP. In the absence of a proton motive force (*pmf*), ATP synthases can hydrolyze ATP and use the energy released to transport protons across the membrane. Because of this ATP hydrolysis activity, ATP synthases are sometimes referred to as rotary ATPases. ATP synthases are classified into two major categories: F-type ATP synthases (F_o_F_1_-ATPase) and V-type ATP synthases (V/A-ATPase) (Figures 1A, B) (Yokoyama and Imamura, 2005; Forgac, 2007; Vasanthakumar and Rubinstein, 2020). F_o_F_1_-ATPases are found in the inner membrane of mitochondria, the thylakoid membrane of chloroplasts, and the plasma membrane of eubacteria (Frasch et al., 2022). V/A-ATPases are present in some eubacteria and archaea and are structurally similar to eukaryotic V-ATPases. The basic structure of both ATPases comprises a rotor complex comprising a hydrophobic c-ring and a stator apparatus surrounding the rotor (Figure 1C). The c-ring in the F_o_/V_o_ domain rotates by the *pmf*, which drives ATP synthesis in the F_1_/V_1_ domain. Conversely, when ATP is hydrolyzed in the F_1_/V_1_ domain and the central rotary axis rotates, protons are transported across the membrane in the F_o_/V_o_ domain.

The isolated hydrophilic F_1_/V_1_ domain exhibits ATP hydrolysis activity and is referred to as F_1_-ATPase and V_1_-ATPase, respectively (Yokoyama et al., 1989; Yokoyama et al., 1990; Weber and Senior, 1997; Suzuki et al., 2016). In the F_1_-ATPase, the catalytic site is located at the interface between the α and *ß* subunits, resulting in three catalytic sites. In V_1_-ATPase, the catalytic site is located at the interface between subunits A and B (Figure 1D) (Maher et al., 2009; Suzuki et al., 2016; Nakanishi et al., 2018). The hydrolysis of a single ATP molecule results in a 120° rotation of the rotor, and the hydrolysis of three ATP molecules results in a full 360° rotation (Imamura et al., 2003).

**FIGURE 2:**
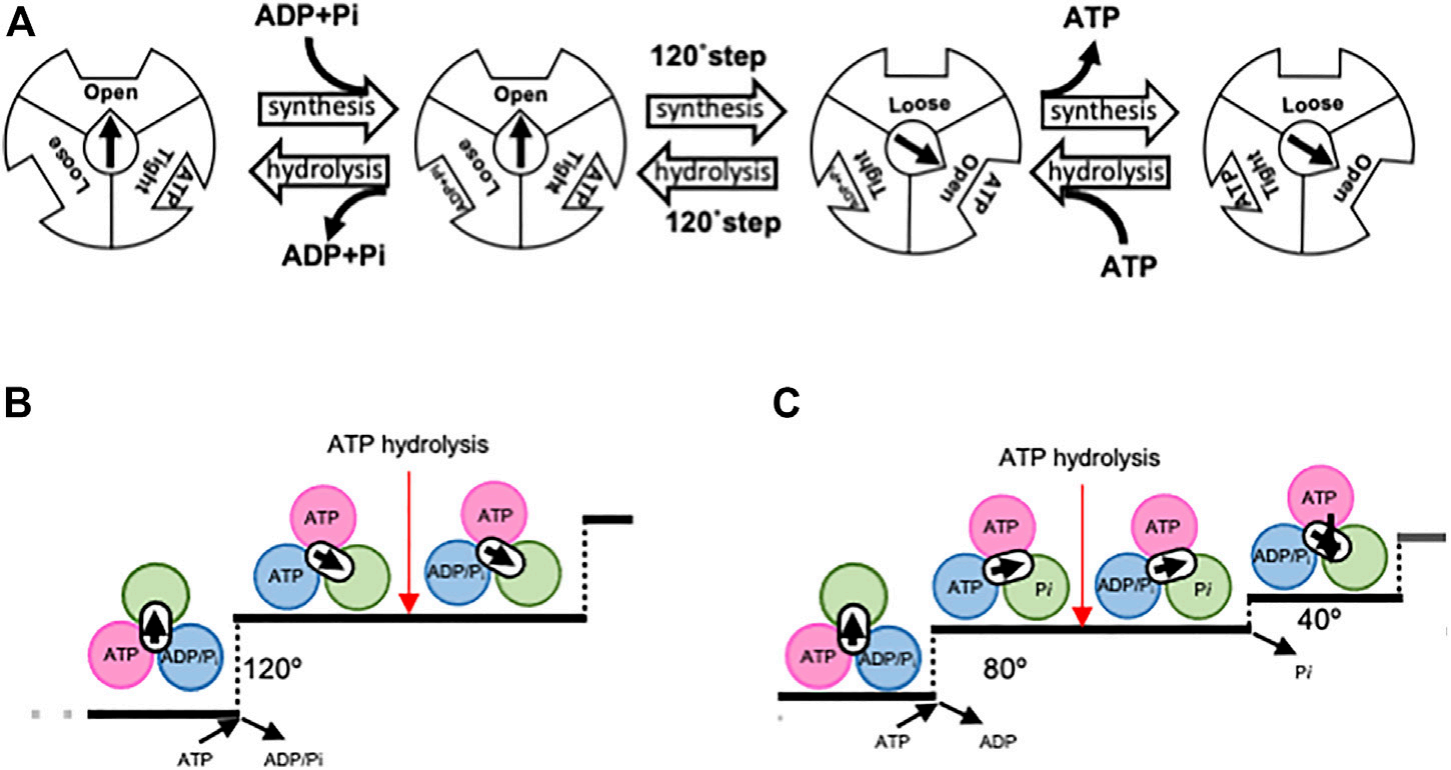
Proposed rotary mechanism of rotary ATPases. Boyer first proposed a model for the binding-change mechanism **(A)**. *Open*, *Loose*, and *Tight* bonds correspond to α_E_β_E_, α_D_β_D_, and α_T_β_T_, respectively. The direction of synthesis is characterized by the binding of ADP and Pi to *Loose*, followed by a 120° step that results in *Tight* becoming *Open* and the release of ATP. Through the conversion of *Loose* to *Tight*, ADP is phosphorylated on *Tight* to form ATP. The reverse sequence of events occurred in the direction of ATP hydrolysis. Proposed chemo-mechanical cycles of V_1_-ATPase **(B)** and F_1_-ATPase **(C)**. Each image was viewed from the V_1_/F_1_ side. In V_1_-ATPase, ATP binding results in a 120° rotation of the DF, followed by ATP hydrolysis. In contrast, F_1_-ATPase has been proposed to operate according to a model in which ATP binding initiates an 80° rotation, followed by ATP hydrolysis and Pi release, leading to a further 80° rotation.

The theory of the binding change mechanism of F_o_F_1_-ATPase rotation was proposed by Boyer over 50 years ago (Boyer et al., 1973). This theory postulates that the three catalytic sites within the F_1_ domain of F_o_F_1_-ATPase sequentially adopt *Loose*, *Tight*, and *Open* forms, with differing nucleotide affinities, as the central γ subunit rotates (Figure 2A). Synchronized changes in the properties of these sites lead to ATP release. The asymmetric hexamer structure of the F_1_ domain predicted by Boyer was confirmed by the crystal structure of bovine heart mitochondrial F_1_-ATPase in 1994 (Abrahams et al., 1994).

The crystal structure of F_1_-ATPase revealed that the three catalytic dimers adopted distinct conformations (Figure 1E). One dimer exhibits an open catalytic interface without any nucleotides (α_E_β_E_), whereas the other dimer displays a closed structure with ADP bound (α_D_β_D_). The third dimer has a partially open structure that accommodates an analog of ATP (α_T_β_T_). Since then, numerous crystal structures of F_1_-ATPase from different species and under different conditions have been reported, but they are essentially identical to those of asymmetric hexamer (Groth and Pohl, 2001; Menz et al., 2001; Kagawa et al., 2004; Cingolani and Duncan, 2011; Rees et al., 2012; Ferguson et al., 2016).

The rotary catalytic mechanism of F_1_-ATPase was directly confirmed by single-molecule observation experiments (Noji et al., 1997). In these experiments, a probe was attached to the rotor γ subunit, whereas the stator α_3_β_3_ was fixed to the glass surface. The rotation of the probe was observed using an optical microscope, and upon ATP addition, the unidirectional rotation of the probe was observed, thereby establishing that F_1_-ATPase functions as a rotary molecular motor driven by ATP. Similarly, the rotary mechanism of the V_1_-ATPase was demonstrated 5 years later by our group (Imamura et al., 2003).

Since the demonstration of the rotation of F_1_-ATPase, numerous single-molecule observation experiments have furthered our understanding of the rotary mechanism of F_1_-ATPase (Yasuda et al., 1998; Yasuda et al., 2001; Shimabukuro et al., 2003; Itoh et al., 2004; Adachi et al., 2007; Furuike et al., 2008; Adachi et al., 2012; Saita et al., 2015). For instance, it has been demonstrated that the binding of one ATP molecule results in a 120° step, which comprises an 80° and 40° substep, with ATP hydrolysis occurring at the 80° position (Figures 2B, C) (Yasuda et al., 2001). In addition, high time-resolution analysis has also been performed on F_1_-ATPase from *Escherichia coli* using tiny gold rods as probes (Spetzler et al., 2006; Hornung et al., 2011). However, the precise mechanism by which the hydrolysis energy of ATP is converted into rotational force remains unclear.

This review aimed to describe the chemo-mechanical coupling mechanism between ATP hydrolysis and rotor rotation revealed by cryo-snapshot analysis of V/A-ATPase. The similarity between V/A-ATPase and F_o_F_1_ suggests that the F_1_ domain shares this mechanism.

## Structure and rotation of V/A-ATPase from *Thermus thermophilus*

The V/A-ATPase from *Thermus thermophilus* is one of the best-studied ATP synthases. It comprises two domains: the V_1_ domain and the V_o_. The V_1_ domain is a rotary motor that rotates its central rotor (DF) within A_3_B_3_, which contains three catalytic sites comprising AB dimers (Yokoyama and Imamura, 2005; Maher et al., 2009) (Figure 1D).

The V_o_ domain (E_2_G_2_ *d*_1_ *a*_1_ *c*_12_) is composed of stator parts, including *a* subunit and two, EG peripheral stalks, and the *d*_1_ *c*_12_ rotor complex (Kishikawa et al., 2020), which comprises a central rotor complex with the DF subunits of V_1_. During the rotation of the central rotor (DF *d*_1_ *c*_12_), caused by the proton motive force, the DF induces changes in the three AB dimers, leading to the cooperative synthesis of ATP from ADP and Pi at the A_3_B_3_ hexamer. The reverse rotation of the central rotor caused by ATP hydrolysis by A_3_B_3_ drives the proton translocation in the V_o_ domain (Yokoyama et al., 1998; Toei et al., 2007).

A single-molecule rotation experiment showed that the V_1_ domain rotates in 120° steps, unlike the 80° steps observed for F_1_-ATPases (Figure 2B). This indicates that ATP binding and hydrolysis in the V_1_ domain occur at the 120° dwell position (Imamura et al., 2005; Furuike et al., 2011). These results were confirmed by a cryo-EM snapshot analysis.

## Structural analysis of V/A- ATPase by cryo-electron microscopy

Although the crystal structures of both F_1_-ATPase and V_1_-ATPase have been determined, no crystal structures of intact V-ATPase or F_o_F_1_ have been reported, owing to the structural variability arising from the rotational state of the F_1_/V_1_ domain.

This situation has changed with the advent of the resolution revolution in cryo-electron microscopy (CryoEM) structural analysis. This has enabled us to obtain the overall structures of F_o_F_1_ and V-ATPases (Allegretti et al., 2015; Zhao et al., 2015; Zhou et al., 2015; Schep et al., 2016; Nakanishi et al., 2018), and further advances in analytical techniques have made it possible to produce high-resolution maps and build atomic models of rotary ATPases in a relatively short period (Hahn et al., 2018; Roh et al., 2018; Gu et al., 2019; Guo et al., 2019; Murphy et al., 2019; Sobti et al., 2019; Abbas et al., 2020).

The single-particle analysis allowed the separation of different structures present in a V/A-ATPase sample through classification into several classes (Cheng, 2018). In yeast V-ATPase, this technique has led to the isolation of three classes with different orientations of the central rotational axis (Zhou et al., 2015). The same approach was applied to V/A-ATPase, resulting in the isolation of three rotational state structures that showed the significant movement of the peripheral hydrophilic domains (EG and the hydrophilic domain of a subunit) in each state.

**FIGURE 3:**
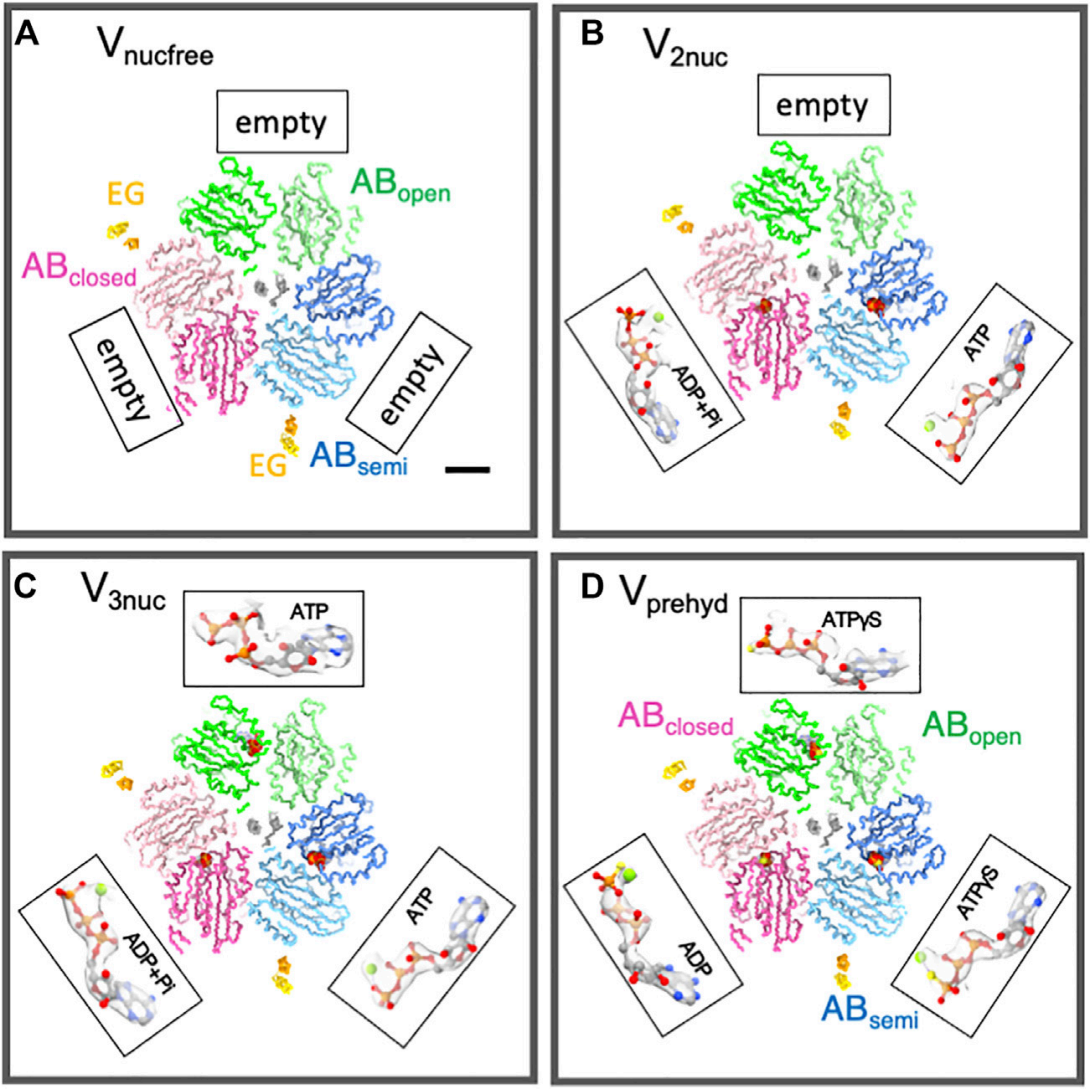
Structures of nucleotide binding sites in each condition. The slice of V_nucfree_ **(A)**, V_prehyd_ **(B)**, V_3nuc_ **(C)**, and V_2nuc_ **(D)** viewed from the V_1_ (cytosolic) side were shown with bound nucleotides. The scale bar is 20 Å. Three catalytic dimers (AB_open_, AB_semi_, and AB_closed_) in each structure are shown in green, blue, and pink, respectively. Two, EG peripheral stalks were shown in yellow. CryoEM maps were represented as semi-translucent. The magnified view of bound nucleotides and Mg ions was highlighted as a ball-and-stick and sphere representation.

To capture the intermediate structure of V/A-ATPase during ATP hydrolysis, we used the TSSA mutant V/A-ATPase, which is less prone to the ADP-inhibited form and has a reduced affinity for ADP at catalytic sites (Nakano et al., 2008). This mutant V/A-ATPase, dialyzed with ethylenediamineetetraacetic acid (EDTA)-containing phosphate buffer to remove bound ADP, was reconstituted on nanodiscs and subjected to cryo-grid preparation. We prepared cryogrids under different reaction conditions, such as waiting for ATP binding with a low ATP concentration, waiting for a catalytic reaction with a saturated ATP concentration, and a saturated ATPγS concentration. These samples were then subjected to cryo-electron microscopy using a Titan Krios. The atomic-resolution structures of the V_1_ domain without nucleotides at the catalytic sites were obtained through single-particle analysis with focused refinement. The nucleotide-free V_1_ structure (V_nucfree_) consisted of an open structure (AB_open_), a slightly closed structure (AB_semi_), and a closed structure (AB_closed_) (Figure 3A). The V_nucfree_ structures were largely similar to the V_1_ structures occupied by nucleotides at three catalytic sites (V_3nuc_), implying that the asymmetry of the V_1_ domain arises from its intrinsic structure rather than conformational changes induced by nucleotide binding. This asymmetric structure of the V_1_ domain has been reported for other V-ATPases (Oot et al., 2016; Suzuki et al., 2016; Abbas et al., 2020; Tan et al., 2022), highlighting the robustness of the V_1_ domain structure.

Subsequently, we performed a single-particle analysis of V/A-ATPase in a reaction solution containing 50 μM or 6 mM ATP, and an ATP regeneration system. Given that ATP binding is the rate-limiting step in the ATP hydrolysis reaction containing 50 μM ATP (which is lower than its *K*_m_ of 500 μM), most of the V/A-ATPase molecules in the reaction solution are in a waiting state for ATP binding. The V_1_ domain structure under these conditions was largely indistinguishable from that of V_nucfree_, comprising AB_open_, AB_semi_, and AB_closed_ structures. Among the catalytic sites, ADP was bound to AB_closed_ and ATP to AB_semi_, whereas AB_open_ had no bound nucleotide (Figure 3B), suggesting that the next ATP molecule should bind to AB_open_. This structure was referred to as V_2nuc_ as it contained two bound nucleotides.

In the presence of 6 mM ATP (>> *K*_m_), the dwell time for ATP binding to the enzyme was negligible, and most of the V/A-ATPase molecules in the reaction solution were in a waiting state for the catalytic reaction after ATP binding. The V_1_ domain under these conditions consisted of AB_open_, AB_semi_, and AB_closed_, all of which were bound to nucleotides (V_3nuc_) (Figure 3C). AB_closed_ was bound to ATP or (ADP + Pi), while ATP binding was also confirmed at both AB_open_ and AB_semi_. Therefore, it is clear that ATP will bind to the AB_open_ in the V_2nuc_, resulting in V_3nuc_ formation. This indicates that the V_3nuc_ represents the state just before the 120° rotation of the rotor takes place. In summary, the 120° rotation does not occur immediately after ATP binds to the V_2nuc_.

Finally, a single-particle analysis of V/A-ATPase was performed in a reaction solution containing 4 mM ATPγS, a slow-hydrolyzing ATP analog. Under these conditions, the reaction solution was substrate-saturated; therefore, most of the resulting structures were those waiting for ATP hydrolysis (V_prehyd_). In V_prehyd_, ATPγS and ADP were observed at all catalytic sites in the V_1_ domain of V/A-ATPase (Figure 3D). Notably, ADP was found at AB_closed_, indicating that ATPγS-bound AB_closed_ had already been hydrolyzed. This also indicated that ATPγS bound to AB_semi_ was waiting for hydrolysis. In the catalytic site of AB_semi_, the amino acid residues responsible for ATP hydrolysis were located further away from the ATP molecule (in this case, ATPγS), resulting in a slower ATPγS-bound AB_semi_ hydrolysis. The hydrolysis of ATPγS bound to AB_semi_ requires a conformational change from AB_semi_ to AB_closed_, which involves 120° rotation in the DF rotor. Thus, V_prehyd_ represents a waiting state for the hydrolysis of ATPγS on AB_semi_ with 120° rotation.

## The rotary mechanism of V/A-ATPase fueled by ATP

The structures of the V_nucfree_ and the nucleotide-containing forms (V_2nuc_ and V_3nuc_) were nearly identical (Figure 3). The asymmetric structure of the V_1_ domain, composed of AB_open_, AB_semi_, and AB_closed_, was maintained regardless of the nucleotide-binding state, suggesting that nucleotide binding to the V_1_ domain does not cause an asymmetric change in its structure. The conformational changes in the V_1_ domain coupled with ATP hydrolysis appear to be discrete, indicating that the power-stroke model, in which the axis rotation is driven by conformational changes in the catalytic subunit, may not be appropriate.

Previous single-molecule rotation experiments on both F_1_-ATPase and V_1_-ATPase indicated that the 120° step (80° step in F_1_) was concurrent with ATP binding, as the histogram analysis of the initiation dwell time indicated a first-order exponential relationship with ATP concentration (Yasuda et al., 2001; Furuike et al., 2011). The presence of the V_3nuc_ structure, in which ATP is bound but the 120° step has not yet occurred, challenges the assumption that ATP binding to the V_1_ domain and the 120° step occur simultaneously.

**FIGURE 4:**
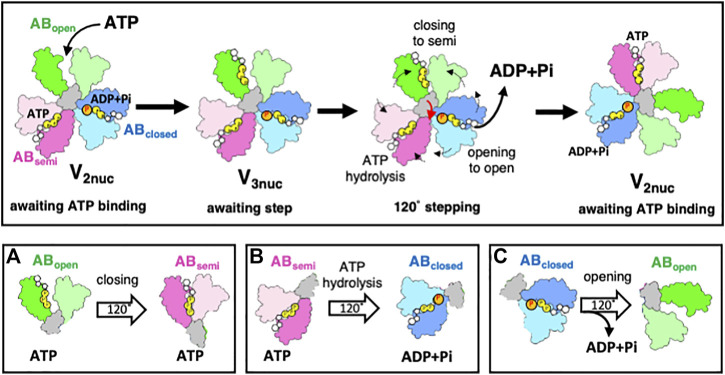
The rotary mechanism of the V_1_ domain powered by ATP hydrolysis. The figure shows schematic models of AB_open_, AB_semi_, and AB_closed_ in green, pink, and blue respectively. The grey region shows the coiled region of the D subunit in contact with A_3_B_3_. In the V_2nuc_ state, waiting for ATP binding, binding of ATP onto AB_open_ does not result in a 120° step. In the V_3nuc_ state, both the closing of AB_open_ and ATP hydrolysis in AB_semi_ result in the opening of AB_closed_, accompanied by ADP and Pi release. The catalytic events in three AB dimers occur simultaneously with a 120° step of DF, leading to a structural transition from V_3nuc_ to V_2nuc_. The lower part of the figure illustrates the simultaneous occurrence of catalytic events at the three catalytic dimers (AB_open_, AB_semi_, AB_closed_) with a 120° step. The events involve changing AB_open_ to AB_semi_ by binding ATP **(A)**. The AB_semi_ also changes AB_closed_ through the hydrolysis of bound ATP **(B)**. AB_closed_ changes AB_open_ through a 120° step of DF, releasing bound ADP and Pi **(C)**.

Structural analysis of V/A-ATPase during rotation provides key insights into the coupling of ATP hydrolysis with the mechanical movement of the rotor. During the transition from V_3nuc_ to V_2nuc_, the three AB dimers undergo the following conformational changes, as depicted in Figure 4: (a) from AB_open_ to AB_semi_, (b) from AB_semi_ to AB_closed_, and (c) from AB_closed_ to AB_open_, which are accompanied by a 120° step in the DF rotor. (a) Is the process by which the AB dimer becomes more closed upon ATP binding. This has been confirmed experimentally, with the *ß*-subunit of F_1_ showing a closed conformation upon ATP binding (Akutsu, 2017). The difference in the nucleotide states bound to AB_semi_ (ATP) and AB_closed_ (ADP and Pi) generates a downhill chemical potential, which drives the 120° step in the process (b) as the AB dimer changes from AB_semi_ to AB_closed_. The release of bound ADP and Pi from AB_closed_ and its conversion to AB_open_ during the process (c) is a result of the interaction between ADP or Pi and the amino acid residues at the catalytic site being cleaved, causing AB_closed_ to open (Kishikawa et al., 2022). This process is non-spontaneous and requires external work. If ADP is bound to AB_closed_, it results in an ADP-inhibited state of the V_1_ domain (Yokoyama et al., 1998; Nakanishi et al., 2018), and the 120° step does not occur even if ATP is bound to AB_open_.

Our model represents an improvement over Boyer’s original model. Unlike Boyer’s bi-site model, which alternates between a state of one and two bound nucleotides and is characterized by a 120° step caused by bound ATP (Figure 2A), the model proposed here is a tri-site model that involves the cooperative interaction of chemical reactions occurring at three distinct sites. This study also sheds light on the role of ATP binding and hydrolysis in the different conformational states of the AB dimer that drives the 120°step of the DF rotor in V/A-ATPase. Both the closure of AB_open_ to AB_semi_ by binding ATP and the hydrolysis of ATP in AB_semi_ trigger the opening of AB_closed_, leading to ADP and Pi release from the catalytic site. This process was synchronized with 120°rotation of the DF rotor. This model highlights the coupling between the downhill chemical potential generated by ATP hydrolysis and the mechanical 120° step of the rotor, as confirmed by the time-resolved structural analysis in the following chapter.

## Time-resolved snapshot structural analysis of V/A-ATPase

**FIGURE 5:**
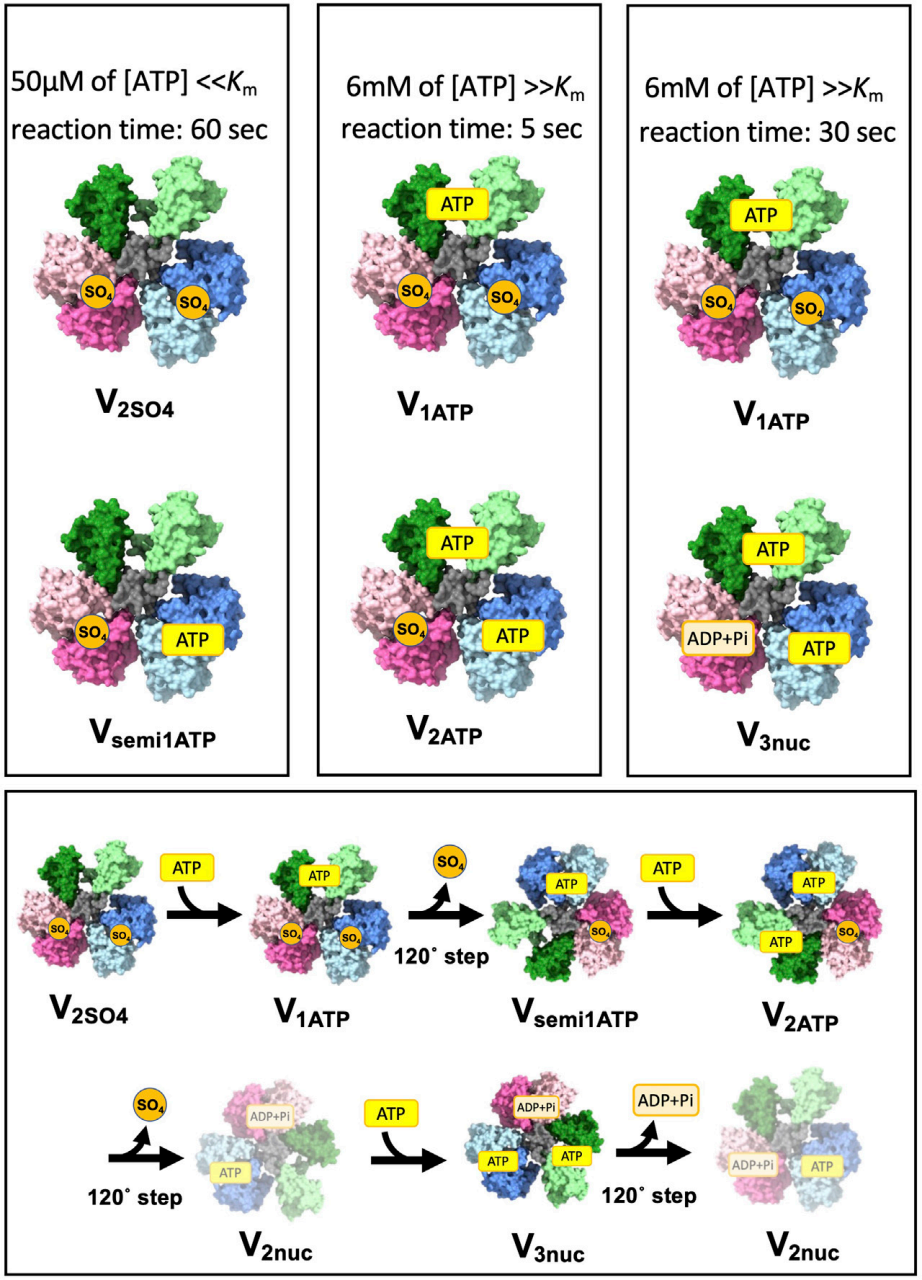
Short-lived initial intermediate V_1_ structure captured by a time-resolved cryo snapshot analysis. This figure shows the results of cryo-electron microscopy (CryoEM) analysis of the structural changes in a nucleotide-depleted V/A-ATPase enzyme upon reaction with different ATP concentrations. The upper columns represent the initial intermediate structures captured at different time points after the reaction with either 50 µM or 6 mM ATP. The lower column shows the structural transition from the initial intermediate structures to the final, steady-state structures (V_2nuc_ and V_3nuc_).

The change in V/A-ATPase from a nucleotide-depleted state to a steady state with nucleotides was studied using the single-particle cryo-EM method. The experiments were performed as shown in Figure 5. V/A-ATPase reacted with a low concentration of ATP and 20 mM sulfate ions for 60 s (Figure 5A), followed by cryo-grid preparation. The resulting structure (V_semi1ATP_) showed ATP binding to AB_semi_ and a 120° rotation, as indicated by the absence of ATP binding to AB_open_. The reaction solution containing V/A-ATPase was then reacted with high ATP concentrations for 5 and 30 s. The 5-s reaction solution yielded two structures, V_1ATP_ and V_2ATP_, with ATP/ADP and sulfate bound to different sites (Figure 5B). V_1ATP_ showed ATP binding to the catalytic site on AB_open_ and sulfate binding to both AB_closed_ and AB_semi_. V_2ATP_ contains two ATP molecules in the catalytic sites on AB_open_ and AB_semi_ and sulfate in the catalytic site on AB_closed_. The 30-s reaction solution yielded two structures, V_1ATP_ and V_3ATP_, with ATP/ADP bound to all three sites, which is similar to the V_3nuc_ obtained under high ATP conditions in a previous study (Figure 5C).

The transition process from the initial state of V_1_ to the V_3nuc_ is shown in Figure 5D. When ATP binds to V_2SO4_, it changes to V_1ATP_. Upon ATP binding to AB_open_, resulting in a 120° step, one molecule of SO_4_ is released, resulting in the transition to V_semi1ATP_. ATP binds to V_semi1ATP_ to produce V_2ATP_. This undergoes a 120° step and releases ADP and Pi, yielding V_2nuc_. ATP binding to the V_2nuc_ produces V_3nuc_. The initially bound SO_4_ is released, and V_1_ eventually reaches a steady state in which V_2nuc_ and V_3nuc_ alternately appear as a result of ATP hydrolysis.

The presence of V_1ATP_ in the 30-s dataset and the absence of V_2ATP_ suggest that an immediate transition from V_2ATP_ to V_3ATP_ occurred. These results suggest that 120° rotation of the rotor does not occur immediately upon the closure of AB_open_ with ATP in the absence of ATP binding on AB_semi_. This is consistent with our model, in which both the closure of AB_open_ with ATP and the hydrolysis of ATP in AB_semi_ drive the open motion of AB_closed_, releasing ADP and Pi coupled with the simultaneous 120° rotation of the rotor.

## Conclusion

The thermophilic F_1_-ATPase has been studied using single-molecule rotation observations, which have shown the presence of at least one intermediate state during the 120° step, in addition to the three rotational states. A recent cryo-electron microscopy (EM) structural study of F_1_-ATPase revealed six structures corresponding to these intermediate states (Sobti et al., 2021). The V_1_ domain comprises only the basic structures of AB_open_, AB_semi_, and AB_closed_, while the βα dimer, the catalytic unit of F_1_-ATPase, adopts intermediate structures beyond these basic structures. This makes the rotational mechanism of the F_1_-ATPase more complex than that of the V/A-ATPase, but the underlying principle remains the same. Our recent structural snapshot analysis of F_o_F_1_ supports this, suggesting that F_o_F_1_ converts the downhill chemical potential generated by ATP hydrolysis into a mechanical step through a similar mechanism (Nakano et al., 2022). This highlights the importance of both ATP binding and hydrolysis in the generation of rotational forces by rotary ATPases.
